# Abundance of Non-Native Birds in the City: Spatial Variation and Relationship with Socioeconomics in a South American City

**DOI:** 10.3390/ani13111737

**Published:** 2023-05-24

**Authors:** Macarena Silva-Ortega, Catalina B. Muñoz-Pacheco, Nélida R. Villaseñor

**Affiliations:** 1 Grupo de Ecología, Naturaleza y Sociedad (GENS), Departamento de Gestión Forestal y su Medio Ambiente, Facultad de Ciencias Forestales y de la Conservación de la Naturaleza, Universidad de Chile, Santiago 8820808, Chilecatalina.munoz.p@ug.uchile.cl (C.B.M.-P.); 2 Escuela de Arquitectura del Paisaje, Universidad Central de Chile, Av. Toesca 1783, Santiago 8370292, Chile; 3 Departamento de Ciencias Químicas y Biológicas, Universidad Bernardo O’Higgins, Av. Viel 1497, Santiago 8370993, Chile

**Keywords:** domestic pigeon, house sparrow, monk parakeet, Santiago de Chile, neighborhood socioeconomic status

## Abstract

**Simple Summary:**

Cities commonly support exotic species that can affect both wildlife and human health, but little is known regarding their distribution across the city and their relationship with socioeconomics. Here, we map the abundance of three non-native birds—domestic pigeon, house sparrow, and monk parakeet—in a Latin American city and investigate the effect of socioeconomics on their abundance. We found the domestic pigeon had a random spatial distribution across the city but reached its greatest abundance in low-income areas. The house sparrow was spatially aggregated in the southern and western areas of the city and reached its greatest abundance in low-income areas. The monk parakeet was spatially aggregated in the northeastern area of the city and reached its greatest abundance in high-income areas. Given that the abundance of non-native birds varies across the city and between socioeconomic groups, species-specific management is needed in different city zones to limit negative effects on native species and prevent human health risks.

**Abstract:**

Cities commonly support a high abundance of non-native species that can affect both wildlife and human health; however, their distribution across the urban environment and their relationship with socioeconomics are not well documented. Here, we map the abundance of three non-native birds in a Latin American city—domestic pigeon (*Columba livia f. domestica*), house sparrow (*Passer domesticus*), and monk parakeet (*Myiopsitta monachus*)—and investigate the effect of socioeconomics on their abundance. We found that *C. livia f. domestica* exhibited a random distribution of abundance across the city but reached its greatest abundance in low-income areas. *P. domesticus* exhibited an aggregated distribution of abundance, being most abundant in the southern and western areas of the city and in low-income areas. *M. monachus* exhibited an aggregated distribution of abundance, being most abundant in the northeastern part of the city and reaching its greatest abundance in high-income areas. Low-income areas likely provide high abundance of food, shelter, and nesting sites for both *C. livia f. domestica* and *P. domesticus,* whereas high income areas have greater tree cover and larger trees in which *M. monachus* can build communal nests. Our study finds that the abundance of non-native birds varies across the city and between socioeconomic groups; therefore, targeted management is needed in different city zones to limit negative effects on native species and prevent zoonotic diseases.

## 1. Introduction

Non-native animals can establish themselves in new ecosystems and negatively affect biodiversity [1]. The main impacts of non-native animals in the wild include competition, predation, herbivory, habitat alteration, disease transmission, and genetic effects [2]. Due to their impacts on ecosystems and the biodiversity they contain, non-native animals are commonly included in the catalogs of invasive species to promote their control [3].

Non-native birds can reach high abundances in urban areas, where these species can be considered pests [4]. Additionally, they can cause a variety of impacts in urban areas, including property damage, noise disturbance, the spread of disease to humans and native species [5], and the displacement of native fauna [6,7]. Despite their high abundance and their varied impacts in urban areas, the effects of non-native birds were previously less investigated than those caused by non-native mammals [8].

A variety of bird species were introduced into the Americas since the conquest, although the largest number of introductions correspond to the 20th century and the last few decades (e.g., [9,10]). Among non-native bird species found in the continent are the domestic pigeon (*Columba livia f. domestica*) and the house sparrow (*Passer domesticus*), which are widely distributed not only in America but around the world [11,12]. The domestic pigeon (*Columba livia f. domestica*), which was domesticated from the rock dove (*Columba livia*) via artificial selection 5000 years ago, is originally from Eurasia and Africa; however, it currently inhabits cities and agricultural fields on various continents [12]. This species has a generalist diet and can reproduce throughout the year [13]. It is a species of interest regarding public health, since it is a vector of more than 30 diseases, such as chlamydiosis, cryptococcosis, aspergillosis, salmonellosis, listeriosis, and staphylococcus infections, which can be transmitted through feces and air [14,15]. The house sparrow is originally from Eurasia and Africa but it was introduced by humans to all continents except Antarctica, and currently inhabits urban areas around the globe [11]. It presents different attributes that explain its success in urban environments. For instance, it is a species with a generalist diet, it displays aggressive behavior towards species of similar or smaller size, it exhibits rapid increases in abundance due to community nesting strategies, and it can colonize new sites due to a high dispersal capacity [16].

On the other hand, there are species from the Americas that were introduced to other countries. The monk parakeet (*Myiopsitta monachus*) is originally from Paraguay, Uruguay, Bolivia, Brazil, and Argentina and was introduced to other counties within the Americas as well as Europe through pet ownership [17,18]. This species can thrive under different environmental conditions has a flexible diet, displays gregarious behavior, and is the only parrot capable of building communal nests without depending on tree cavities or cliffs [18]. It interacts with non-native birds (e.g., forages with house sparrows, shares nests with both house sparrows and domestic pigeons, etc.), which can increase the risk of pathogen transmission [19]. It is a species that can be considered a pest due to big losses in agriculture. For instance, in Argentina, the monk parakeet causes crop losses worth more than US$ 1 billion per year [20].

Since non-native species cause environmental and economic impacts, as well as risks to human health, it is important to understand the distribution of birds in the city and identify whether some social groups are more exposed to their impacts. Although there is growing evidence on the relationship between socioeconomic level and species diversity [21], with native species tending to be more abundant in sites with higher economic income [22,23], there is a lack of studies on how socioeconomic factors relate to the abundance of exotic species (e.g., [24]).

To provide scientific evidence that contributes to making decisions on the management of non-native species in cities, we evaluate the abundance of three introduced species in Santiago de Chile. We aim to map the abundance of each species in the city and analyze their abundance according to the socioeconomic level of different neighborhoods. Based on the results and the international literature, we discuss the factors that may influence abundance patterns, as well as the effects on urban ecosystems and the human population.

## 2. Materials and Methods

### 2.1. Study Area

The study was located in the city of Santiago de Chile, the capital of Chile. The city has an area of ~800 km^2^ and is the home of more than 6 million people, which corresponds to 35% of the national population [25]. The city is in Central Chile, which is an area with high level of endemism in flora and fauna due to geographic isolation caused by natural barriers [26]. The landscape is now strongly modified due to land use change for both agricultural and urban purposes [27]. This high level of endemism, together with the loss of natural ecosystems, positions Central Chile as a priority site for conservation (being a biodiversity hotspot) [28]. The climate in the city of Santiago de Chile is Mediterranean, with average annual precipitation of 304 mm [25], which concentrates in the coldest period (winter), with dry conditions predominant during the summer period [29]. The average annual temperature is 15 °C, with −2.5° being the minimum temperature in winter and 35.5 °C being the maximum temperature in summer [25].

### 2.2. Selection of Sampling Sites

**Figure 1:**
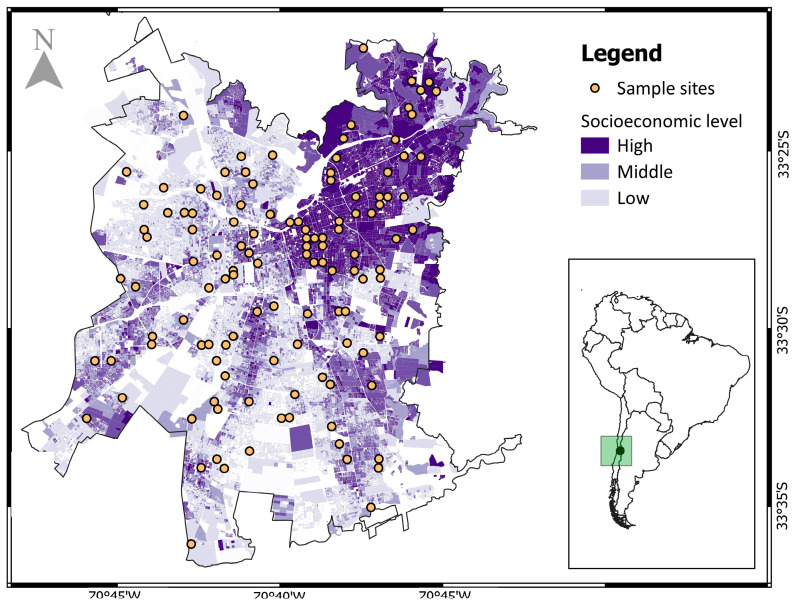
Distribution of sampling sites and socioeconomic levels in Santiago de Chile. Bottom right panel shows the location of the city with respect to South America.

Sampling sites corresponded to 120 sites located in residential areas with different socioeconomic levels, which were defined in previous research [23]. For the selection of the sites, the authors identified three socioeconomic groups in Santiago de Chile—high: higher college education and household income greater than US$28,800 per year; medium: technical or secondary education and family income greater than US$13,200 per year; and low: less schooling and average family income less than US$8400 per year; [30]. A stratified random selection approach was used to select 20 sites in residential areas for each combination of socioeconomic status (three levels) and distance from the urban limit (border and interior), resulting in 120 sites [23] (Figure 1).

### 2.3. Bird Counts

We recorded birds at each site using point counts. All counts were carried out in the southern hemisphere autumn season (28 April to 16 May) and the southern hemisphere winter season (20 July to 6 August) in 2021. The counts were conducted in autumn and winter since, in Mediterranean climates, autumn is a transitional season with moderate temperatures and increasing rainfall, while winter is colder, wetter period, with more frequent and intense rainfall [31]. These environmental modifications can generate changes in the composition of bird species due to migration [32] and food availability [33]; however, few prior urban studies focused on these seasons [34]. In each season, all sites were visited by two observers on different days, who recorded all birds seen or heard within 5 min in a radius of 50 m [23,35]. Thus, at the end of the sampling period, we obtained four counts per site. All data were collected during the morning, from 6:00 to 11:57 a.m., to coincide with a period of high bird activity.

### 2.4. Data Analysis

The abundance of each non-native bird was mapped in the city to observe the sectors with highest abundance. Firstly, for each of the three introduced species (*P. domesticus*, *C. livia f. domestica* and *M. monachus*), the accumulated abundance per sampling site was calculated by season. The inverse distance weighting (IDW) method was then used in QGIS, which corresponds to a deterministic spatial interpolation that assumes that the closest sites are more similar than those that are further away [36]. This method was used to visualize patterns of richness and abundance in both plants and animals [37,38,39]. With this interpolation method, it was possible to control the importance of known sites on the interpolated values (power (p)), as well as sites that can influence the interpolation (neighborhood (n)). Recommended values were used, where *p* = 2 and *n* = 12 [40]. To aid the detection of changes in abundances between seasons, for each species, we calculated the difference in abundance between autumn and winter at each site and interpolated the differences [41]. To assess species’ spatial autocorrelation, we used the Moran’s index that assesses whether the abundance of a species is aggregated, dispersed, or random [42]. 

We evaluated the influence of each neighborhood’s socioeconomic level and the season on the abundance of each non-native species. For this, Generalized Linear Mixed Models (GLMM) with Poisson distribution were fitted, which described the abundance of each species according to the socioeconomic level (high, medium, low) and the season (autumn, winter) using “lme4” package [43] in R.3.4.4 [44]. The response variable was the accumulated abundance of a species per sampling site at each season (two counts). The natural logarithm of counts was used as an offset to consider differences in sampling effort (because one site had only one count). The sampling site was included as a random effect (*n* = 120) [45]. 

## 3. Results

A total of 479 bird counts were performed, from which we obtained 3504 records of non-native birds. Of these records, 52% correspond to domestic pigeon, 35% correspond to house sparrow, and 13% correspond to monk parakeet. Thus, the most abundant species was domestic pigeon, followed by house sparrow and, finally, monk parakeet, with similar values recorded between seasons (Table 1).

**Figure 2:**
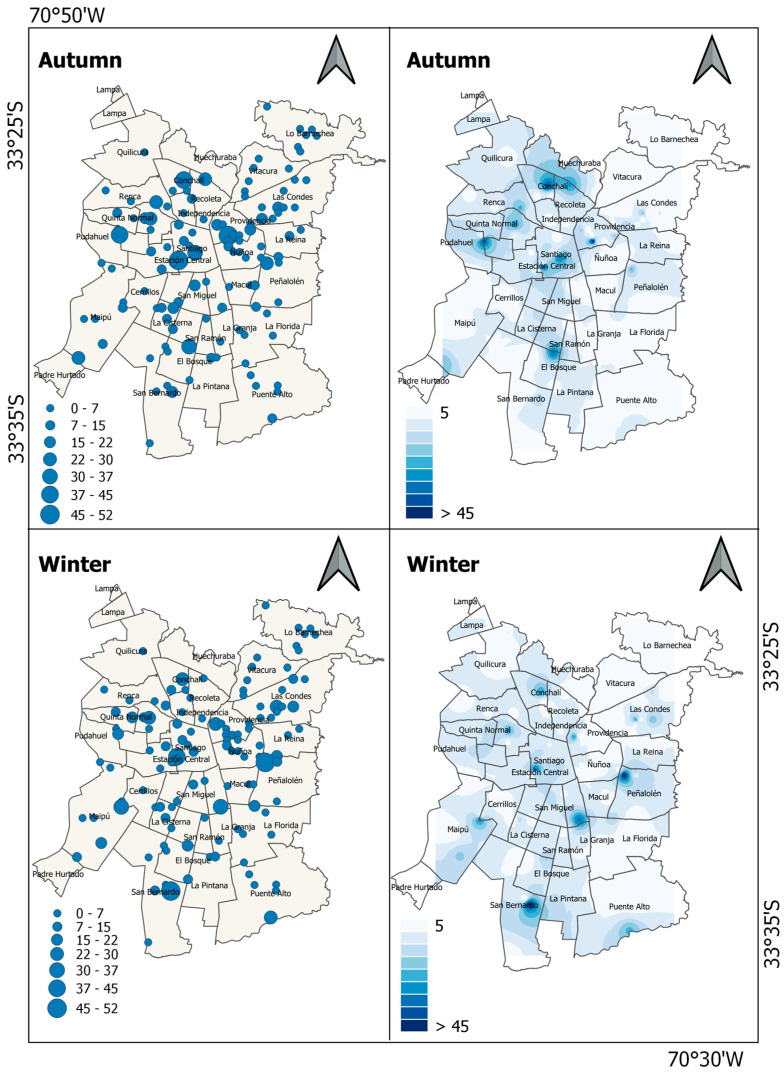
Maps showing accumulated abundance of domestic pigeon (*C. livia f. domestica*) recorded at sampling sites across Santiago de Chile and abundance estimation of domestic pigeon using IDW interpolation.

**Figure 3:**
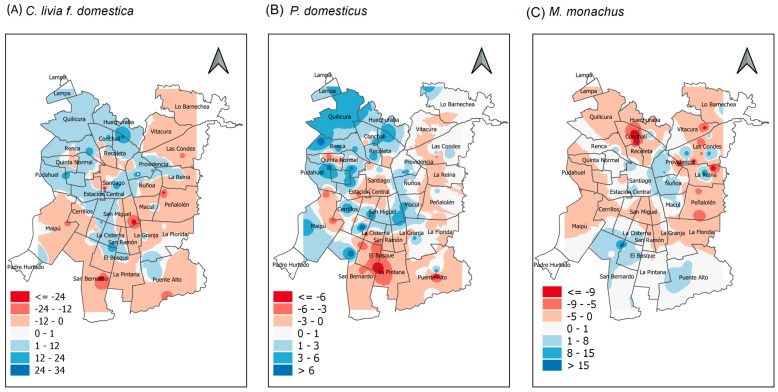
Maps showing difference in abundance for (**A**) domestic pigeon (*C. livia f. domestica*), (**B**) house sparrow (*P. domesticus*), and (**C**) monk parakeet (*M. monachus*) across Santiago de Chile, estimated via IDW interpolation. Blue areas show that a species was more abundant in autumn than winter, whereas red areas show that a species was more abundant in winter than in autumn.

Maps showing the spatial variation in the abundance of the domestic pigeon in Santiago de Chile lack a clear pattern (Figure 2), although the species was more abundant in the north–central zone in autumn, whereas in winter, there were foci of high abundance in different zones (Figure 3A). Moran’s index revealed that the abundance of the domestic pigeon presents random distribution in both autumn and winter (autumn: Moran’s index = 0.01, z-score = 0.44, *p* = 0.66; winter: Moran’s index = −0.0001, z-score = 0.23, *p* = 0.82).

**Figure 4:**
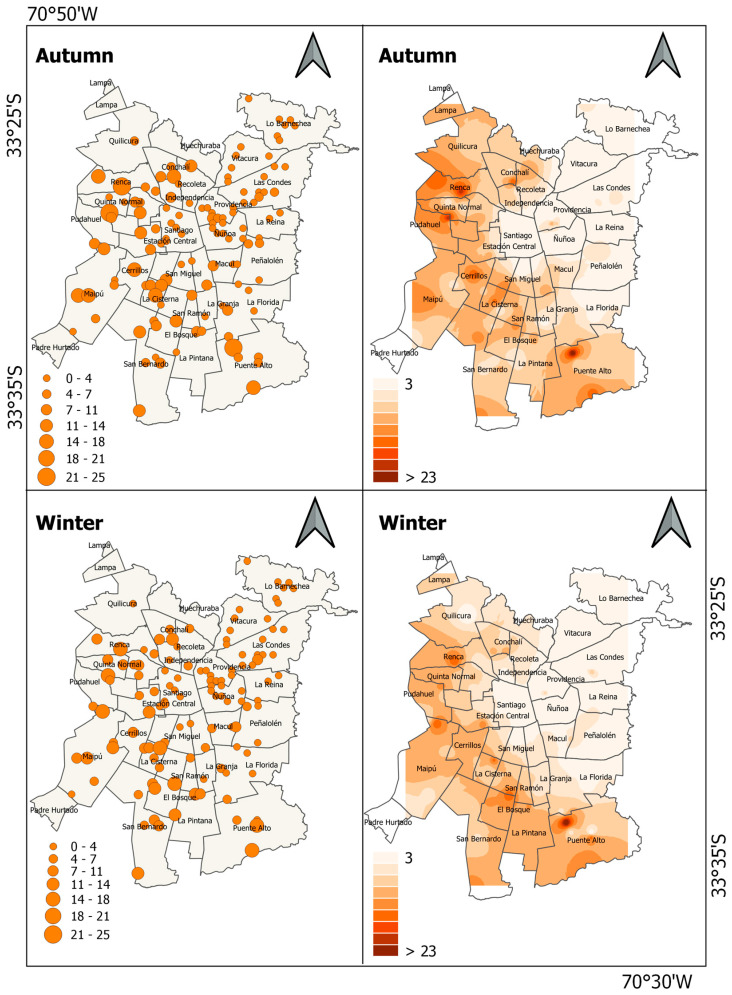
Maps showing accumulated abundance of house sparrow (*P. domesticus*) recorded at sampling sites across Santiago de Chile and abundance estimation of house sparrow using IDW interpolation.

Maps showing the spatial variation in the abundance of the house sparrow in Santiago de Chile show that the species was more abundant in the western and southern zones, whereas central and eastern zones exhibited low abundance (Figure 4). When comparing the distribution of abundance between seasons, the species was more abundant in the northern zone in autumn than in winter (Figure 3B). Moran’s index revealed that the abundance of the house sparrow was spatially aggregated in both seasons (autumn: Moran’s index = 0.35, z-score = 10.07, *p* < 0.001; winter: Moran’s index = 0.40, z-score = 11.38, *p* < 0.001).

**Figure 5:**
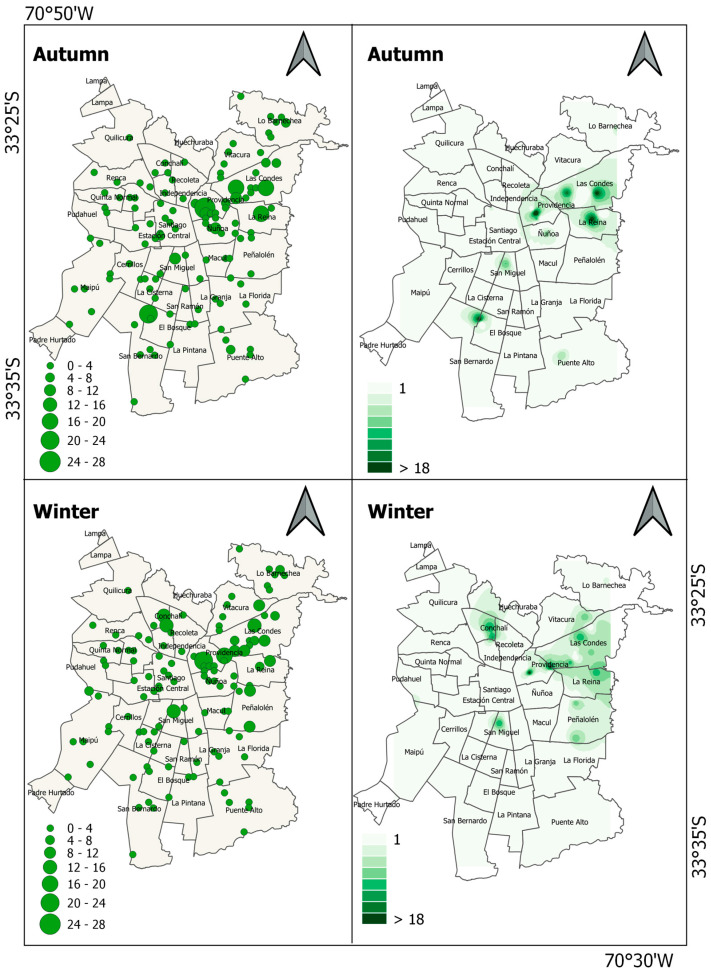
Maps showing accumulated abundance of monk parakeet (*M. monachus*) recorded at sampling sites across Santiago de Chile and abundance estimation of monk parakeet using IDW interpolation.

The monk parakeet exhibited high abundance in the northeastern zone of Santiago de Chile in both seasons (Figure 5). There were several foci of higher abundance in winter than in autumn, especially in the eastern and northern areas of the city (Figure 3C). Moran index revealed that the abundance of monk parakeet was spatially aggregated in both seasons (autumn: Moran’s index = 0.08, z-score = 2.57, *p* < 0.05; winter: Moran’s index = 0.15, z-score = 4.49, *p* < 0.001).

**Figure 6:**
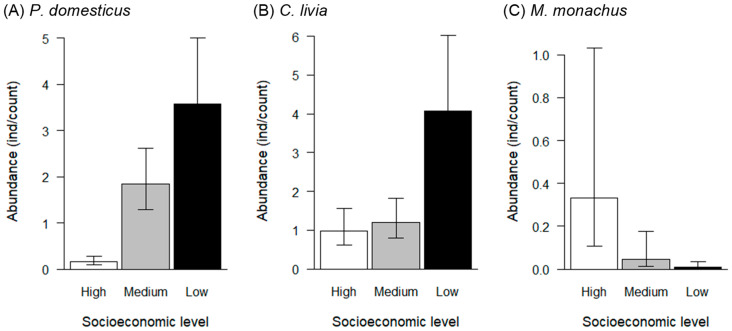
Mean abundance (individuals/count) of domestic pigeon (*C. livia f. domestica*), house sparrow (*P. domesticus*), and monk parakeet (*M. monachus*) by socioeconomic level, estimated using Generalized Linear Mixed Models in autumn. Error bars show 95% confidence intervals.

Generalized Linear Mixed Models evidenced significant effects of each neighborhood’s socioeconomic level, but not season, on the abundance of non-native species. The domestic pigeon’s abundance was low in sites located in neighborhoods at high and medium socioeconomic levels, while it was significantly higher in neighborhoods at a low socioeconomic level (*p* < 0.001) (Figure 6A, Table 2). We did not find a significant effect of season on the domestic pigeon’s abundance (*p* = 0.86, Table 2). The abundance of the house sparrow was very low in sites located in neighborhoods at a high socioeconomic level and significantly more abundant in neighborhoods at medium and low socioeconomic levels (*p* < 0.001) (Figure 6B, Table 2). The house sparrow’s abundance was lower in winter than in autumn, although the difference was not statistically significant (*p* = 0.056, Table 2). Finally, the monk parakeet’s abundance was significantly higher in neighborhoods at a high socioeconomic level than in neighborhoods at medium and low socioeconomic levels (*p* < 0.05), and there was no effect of season (Figure 6C, Table 2).

## 4. Discussion

This research shows that the abundance of non-native bird species varies across the city and changes significantly based on a neighborhood’s socioeconomic level. The domestic pigeon and the house sparrow are abundant in low-income neighborhoods, unlike the monk parakeet, which is abundant in neighborhoods with greater economic resources. Due to different abundances of non-native bird species across the city, the ecosystems, wildlife, and human populations will be differentially exposed to their impacts.

From the studied bird species, the two most abundant urban bird species were the domestic pigeon (*C. livia f. domestica*) and the house sparrow (*P. domesticus*). The dominance of these two non-native birds agrees with previous research in the city of Santiago de Chile (e.g., [35,46,47]) as well as in other urban environments in Latin America [48,49,50,51], and North America [52,53]. Although these species commonly dominate urban communities, they can have declining populations in their native distribution range [54,55]. For instance, the house sparrow exhibits a dramatic population decline, starting in the second half of the 20th century, in Europe [55,56]. Its decline might be due to changes in urban environmental conditions, such as the change from horses to automobiles that decreased food supply and increased mortality, and the modification of housing that decreased nesting sites, affecting reproductive success [57,58]. In contrast, domestic pigeons (*Columba livia f. domestica*) have good reproductive success in their natural distribution and worldwide, unlike its ancestral variety, the Rock Pigeon (*Columba livia*), whose populations are declining [54,57].

The monk parakeet was the third most abundant exotic species in our study area. Its population size is rapidly increasing in urban areas where the species was introduced [59,60], a trend that might be due to nesting structures and food availability [59]. In addition, the species’ productivity, recruitment, and survival rates are higher in its invasive range, possibly due to the lack of natural predators [61]. Although the monk parakeet can be abundant in cities in its invasive range, it is surprising that it is not a common urban bird in its native range [48,50,62], where it mainly inhabits forests, scrublands, savannahs, and rural areas [63,64]. It would be interesting to understand the mechanisms that explain the differences, such as whether genetic changes occurred in their invasive populations that affect behavior and reproduction, since genetic changes were previously observed in invasive species [65].

The domestic pigeon is a synanthropic species that commonly exhibits its greatest abundance in the city center [66]; however, we did not find this pattern. We found a significant effect of neighborhood socioeconomics, with the species being more abundant in neighborhoods at a low socioeconomic level. Neighborhoods where people of low socioeconomic level live commonly exhibit high human population density, high housing density, large urban waste, and low vegetation cover, which are all attributes that are associated with a higher abundance of the domestic pigeon [35,67,68]. These environmental characteristics favor the species, since it is a generalist and opportunist species that can feed on human waste and nest, refuge, and perch in buildings and urban infrastructure [68,69].

The house sparrow was more abundant in the western and southern zones of the city, being more abundant in residential areas at a lower socioeconomic level. Similar results were previously found in Europe, where house sparrows are more common in areas of relatively low socioeconomic status and are almost completely absent from areas of high socioeconomic status [58]. Different characteristics of the species explain these results. Firstly, house sparrows often nest in cavities associated with the roofs of low buildings, such as houses, and avoid new buildings [58]. This finding is consistent with the residential areas located in the western and southern zones of the city, which are composed mainly of single-story housing up to four-story buildings, where people of medium and low socioeconomic status reside [70]. In contrast, low house sparrow abundance was found in the central and eastern areas of the city, probably because high-rise buildings are concentrated in the city center, while the eastern zone is dominated by new and high-rise buildings, as well as neighborhoods at a high socioeconomic level with large properties that result in a low housing density [71]. Secondly, neighborhoods at a lower socioeconomic level in Santiago de Chile present less tree cover and greater impervious surface [72,73], which commonly relate to greater abundance of the species [35]. Finally, unmanaged herbaceous vegetation is more frequent in areas at a lower socioeconomic level [74]; this type of vegetation, in combination with bare ground and impervious surfaces, would provide habitat conditions that contribute to the feeding efficiency of sparrows [75,76].

The monk parakeet was more abundant in the northeastern zone of Santiago de Chile, which concentrates the neighborhoods at a higher socioeconomic level. Neighborhoods where people with higher incomes live have greater tree cover [23,41], providing an important resource for this species to build voluminous communal nests [77]. In neighborhoods at a lower socioeconomic level, it is possible that there is a lower number of tall trees and therefore, a lower abundance of monk parakeets [18]. In Spain, this exotic species lives mainly in urban parks with large trees, which could help reduce predation [78]. In Chile, the species was initially recorded in the wild in the eastern zone of Santiago de Chile [20]; however, due to its high invasive potential, the species rapidly expanded across the city, establishing reproductive colonies, and achieving massive dispersal along the country [17].

Differences in the abundance of non-native bird species across the city, according to socioeconomics, suggest that these species differentially affect native communities and people in the city. Given that areas at a lower socioeconomic level have higher population densities [79], more people would be exposed to the impacts derived from domestic pigeons and house sparrows. In contrast, residential areas at a high socioeconomic level would be more exposed to the impacts derived from the invasion of the monk parakeet. Some of the impacts of the domestic pigeon include the transmission of parasites, bacteria, and viruses that affect health [80], with this species hosting more than 60 pathogenic micro-organisms that affect humans [81]. In the case of the house sparrow, it was found that sparrow-invaded sites had lower native bird species richness than non-invaded areas [16]. House sparrows also present diseases that can be transmitted to wildlife and people. For example, sparrows worldwide have 8–13% prevalence of *Toxoplasma gondii* [82,83], a protozoan parasite that infects birds and mammals throughout the world, including humans [84]. In the case of the monk parrakeet, it presents protozoa (*Cryptosporidium* sp.), bacteria (*Chlamydia psittaci*), and mites (*Mesostigmata*) that cause diseases in native birds and have zoonotic potential [85]. In addition, the monk parrakeet competes with granivorous birds and exhibits aggressive behaviors against other species [86]. Although these behaviors could affect native species in the northeastern area of Santiago de Chile, they can also provide nesting sites for secondary cavity-nesting species [19]. In fact, during the field surveys, a pair of kestrels (*Falco sparverius*) were observed using an apparently abandoned monk parakeet nest. 

Our study shows the consistency of results in autumn and winter seasons. Most studies of urban birds in Latin America were performed in spring or summer season, with few studies in autumn and winter [34]. Studies in these seasons are relevant, since several native birds find refuge in the city during the non-breading season, and migratory birds can arrive in cities during migration and the winter season [23,87,88]. Future research could investigate patterns throughout the year or compare reproductive and non-reproductive seasons [34]. 

Finally, non-native species require targeted management strategies. Given that domestic pigeon and house sparrow are more abundant in sites with low woody vegetation cover, habitat management should involve increasing tree and shrub cover to limit their abundance in low socioeconomic status areas [35,89,90]. In contrast, monk parakeet is more abundant in areas with high vegetation cover, especially where there are large trees to build communal nests; therefore, avoiding the provision of optimal trees for nesting [18] could contribute to limiting its abundance in high socioeconomic status areas. Other methods for controlling invasive populations in urban areas include removal of domestic pigeons using cage trapping to decrease population sizes after two months of extraction [91], and fertility control drugs in food that lead to population reductions of 50 to 70% after four years [92,93]. Methods for the control of house sparrow include sterilized seeds, removal using mist nets, and the removal of nests and chicks, which result in a population reduction of 87% [89]. For monk parakeet, there is a lack of safe and effective methods for controlling populations, although the use of Diazacon provided in seeds contributes to inhibiting reproduction [94,95]. When aiming to manage non-native species, it is important to consider territorial and social aspects [18,93] to effectively prevent zoonotic diseases that are harmful to humans and promote a more biodiversity-sensitive city.

## 5. Conclusions

Our study shows that three non-native species—domestic pigeon, house sparrow, and monk parakeet—differ in their abundance patterns in Santiago de Chile. The domestic pigeon had different foci of high abundance, which were consistent with a random pattern, but reached its highest abundance in neighborhoods at a low socioeconomic level. The house sparrow had greater abundance in western and southern zones of the city, which was consistent with an aggregated pattern, and reached a greater abundance in neighborhoods at a low socioeconomic level. The monk parakeet was abundant in the northeastern zone of the city, which was consistent with an aggregated pattern, and had its highest abundance in neighborhoods at a high socioeconomic level. Given that wildlife from different zones and people of different socioeconomic groups are likely to be interacting with non-native birds to different degrees, targeted management is needed to limit their impacts on humans, wildlife, and ecosystems.

## Figures and Tables

**Table 1:** Non-native bird species recorded in city of Santiago de Chile. Average abundance and standard error recorded via count in autumn and winter seasons are shown.

Family	Common Name	Scientific Name	Autumn	Winter
Columbidae	Domestic pigeon	*Columba livia f. domestica*	3.7 ± 0.5	3.1 ± 0.4
Passeridae	House sparrow	*Passer domesticus*	2.7 ± 0.2	2.4 ± 0.2
Psittacidae	Monk parakeet	*Myiopsitta monachus*	0.9 ± 0.2	0.9 ± 0.2

**Table 2:** Estimated parameters for abundance of domestic pigeon (*C. livia f. domestica*), house sparrow (*P. domesticus*), and monk parakeet (*M. monachus*) according to Generalized Linear Mixed Models.

Species	Predictive Variables	Estimated Coefficient	Standard Error	*p*-Value	
*C. livia f. domestica*	Intercept	−0.02	0.23	0.940	
	Socioeconomic _Medium_	0.20	0.31	0.510	
	Socioeconomic _Low_	1.42	0.30	<0.001	***
	Season _Winter_	0.01	0.04	0.868	
*P. domesticus*	Intercept	−1.87	0.30	<0.001	***
	Socioeconomic _Medium_	2.48	0.33	<0.001	***
	Socioeconomic _Low_	3.15	0.34	<0.001	***
	Season _Winter_	−0.11	0.06	0.056	.
*M. monachus*	Intercept	−1.10	0.58	0.057	.
	Socioeconomic _Medium_	−2.01	0.79	0.011	*
	Socioeconomic _Low_	−3.76	0.85	<0.001	***
	Season _Winter_	0.03	0.09	0.778	

*p*-value: . <0.1; * <0.05; *** <0.001.

## Data Availability

Data will be made available on request.
